# WISP-1 drives bone formation at the expense of fat formation in human perivascular stem cells

**DOI:** 10.1038/s41598-018-34143-x

**Published:** 2018-10-23

**Authors:** Carolyn A. Meyers, Jiajia Xu, Greg Asatrian, Catherine Ding, Jia Shen, Kristen Broderick, Kang Ting, Chia Soo, Bruno Peault, Aaron W. James

**Affiliations:** 1 Department of Pathology, Johns Hopkins University, Baltimore, 21205 United States; 2 Division of Growth and Development and Section of Orthodontics, School of Dentistry, UCLA, California, Los Angeles 90095 United States; 3 Department of Plastic Surgery, Johns Hopkins University, 21205 Baltimore, United States; 4 UCLA and Orthopaedic Hospital Department of Orthopaedic Surgery and the Orthopaedic Hospital Research Center, California, Los Angeles 90095 United States; 5 Division of Plastic and Reconstructive Surgery, Department of Surgery, David Geffen School of Medicine, University of California, California, Los Angeles 90095 United States; 6 Center For Cardiovascular Science and MRC Center for Regenerative Medicine, University of Edinburgh, Edinburgh, United Kingdom

## Abstract

The vascular wall within adipose tissue is a source of mesenchymal progenitors, referred to as perivascular stem/stromal cells (PSC). PSC are isolated via fluorescence activated cell sorting (FACS), and defined as a bipartite population of pericytes and adventitial progenitor cells (APCs). Those factors that promote the differentiation of PSC into bone or fat cell types are not well understood. Here, we observed high expression of WISP-1 among human PSC *in vivo*, after purification, and upon transplantation in a bone defect. Next, modulation of WISP-1 expression was performed, using *WISP-1* overexpression, WISP-1 protein, or *WISP-1* siRNA. Results demonstrated that WISP-1 is expressed in the perivascular niche, and high expression is maintained after purification of PSC, and upon transplantation in a bone microenvironment. *In vitro* studies demonstrate that WISP-1 has pro-osteogenic/anti-adipocytic effects in human PSC, and that regulation of BMP signaling activity may underlie these effects. In summary, our results demonstrate the importance of the matricellular protein WISP-1 in regulation of the differentiation of human stem cell types within the perivascular niche. WISP-1 signaling upregulation may be of future benefit in cell therapy mediated bone tissue engineering, for the healing of bone defects or other orthopaedic applications.

## Introduction

The vascular wall within adipose tissue is a source of mesenchymal stromal progenitors, often referred to as perivascular stem/stromal cells (PSC), vascular wall resident mesenchymal stem cell (MSC), or tissue-specific MSC. Adipose tissue is an appealing source of stromal cells for skeletal regenerative medicine, as it is an easily accessible and dispensable cell source^1–3^. The stromal vascular fraction (SVF) of adipose tissue has been previously used for bone repair, but formed bone tissue unreliably^4^ or with a low efficacy^5^. As an alternative cell source, PSC from subcutaneous white adipose tissue are an uncultured, fluorescence activated cell sorting (FACS) derived cell population, and are defined as a bipartite population of CD146+CD34−CD45−CD31− pericytes and CD34+CD146-CD45-CD31- adventitial progenitor cells (APCs)^6,7^. Although their location and antigen expression differ, pericytes and APCs have conserved and overlapping pro-osteogenic/pro-vasculogenic properties in the context of bone tissue engineering (see^8^ for a review). Both perivascular cell populations express characteristic MSC markers *in situ*, after FACS purification, and after *in vitro* expansion (including for example CD44, CD73, CD90, and CD105)^9,10^. In comparison to cells from the SVF of the same patient sample, PSC have shown significantly greater potential for bone formation by their ability to form bone in an intramuscular location^7,11^, calvarial defect model^12^, or rat spinal fusion model^6,11^. However, those factors that maintain quiescence or conversely promote the differentiation of PSC into bone or fat cell types are not well understood.

Our prior studies identified *WISP-1* (WNT1-inducible-signaling pathway protein 1) as a novel factor highly upregulated among human PSC (72 fold increase in comparison to unpurified stromal vascular fraction by RNA Sequencing). WISP-1 is a CCN (Cysteine-rich angiogenic inducer 61 [Cyr61], Connective tissue growth factor [CTGF], Nephroblastoma overexpressed [Nov]) family member which to our knowledge has not been described in a perivascular location. WISP-1 is better known to be expressed in osteoprogenitor cells, either during skeletal development or fracture repair^13^. CCN family members all have roles in osteochondral cell specification, although the relative importance for bone or cartilage differentiation differs between family members^14–16^. Mechanistically, WISP-1 exerts complex and incompletely understood effects on both canonical Wnt and BMP (Bone morphogenetic protein) signaling in order to specify MSC lineage determination and osteogenic differentiation^13,17–19^. For example, at the extracellular surface of the MSC, WISP-1 binds to BMP2 to enhance BMP2 binding to BMPR1/2, resulting in Smad1/5/8 phosphorylation and canonical BMP signaling activation^18^. Recent studies have also found WISP-1 to functionally ‘de-repress’ canonical Wnt signaling, by blocking Sclerostin (SOST) binding to LRP5^19^. The exact mechanism by which WISP-1 blocks SOST/LRP5 binding is not yet known. As well, recent studies have elucidated important roles for WISP-1 in bone maintenance. Mice with global *Wisp-1* deficiency display a low bone mass phenotype, with reduced cortical and trabecular bone, reduced osteoprogenitor cell differentiation, increased osteoclast activity, and increased sensitivity to ovariectomy induced bone loss^19^. Conversely, *Wisp-1* overexpression driven by the Col1a1 promoter leads to a high bone mass phenotype^18^. In aggregate, WISP-1 is a novel pro-osteogenic secreted matricellular protein that enhances both Wnt and BMP signaling. These observations led us to examine the localization and function of WISP-1 within the perivascular niche and in human PSC.

## Results

### WISP-1 localization to the perivascular niche

**Figure 1:**
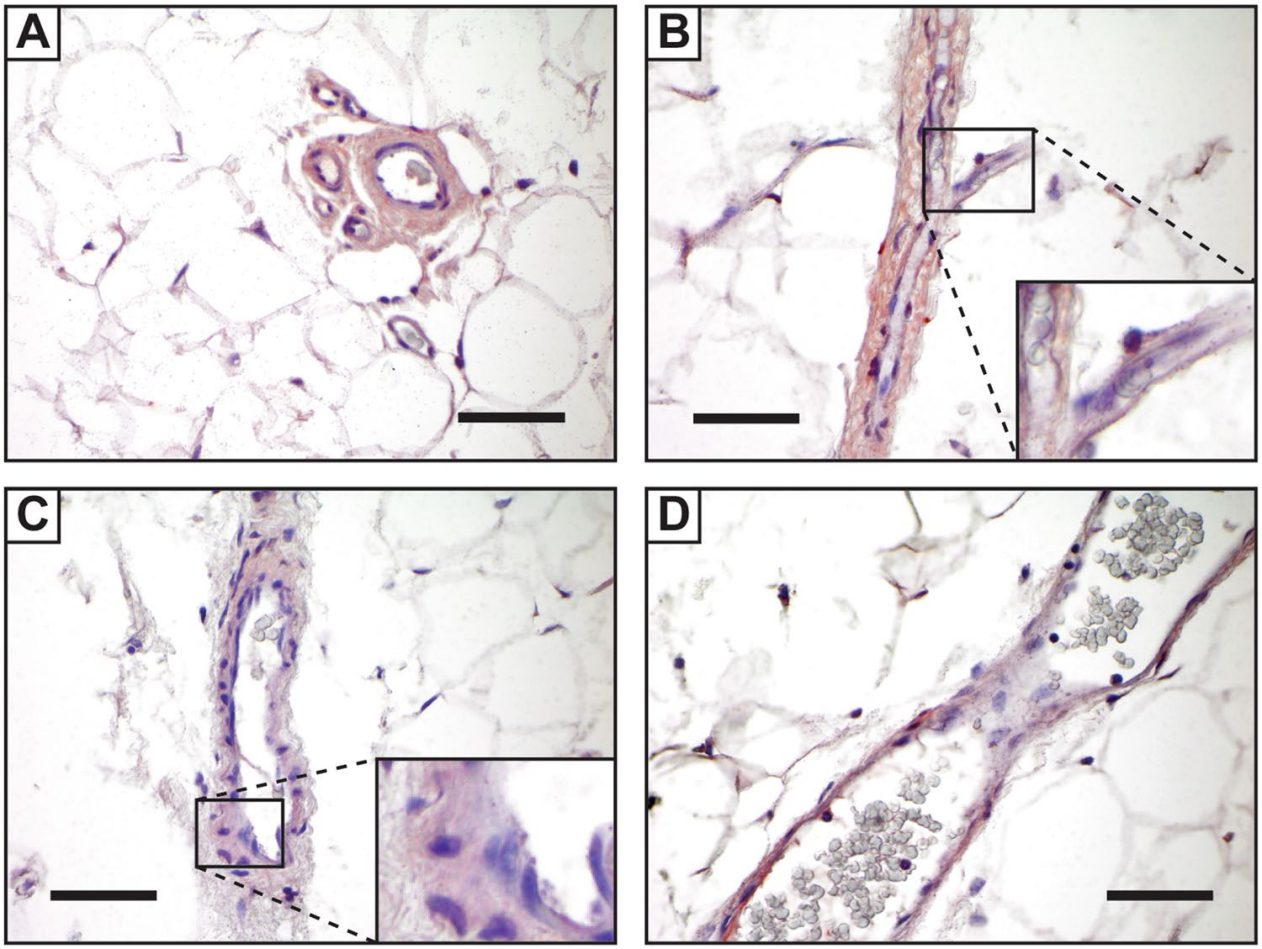
Immunohistochemical detection of WISP-1 protein in human adipose tissue. (**A)** WISP-1 immunoreactivity within the vascular wall of small caliber vessels in cross section. (**B**) WISP-1 immunoreactivity within a venule (center) and branching capillaries in longitudinal cross section. (**C**,**D**) WISP-1 immunoreactivity in larger caliber veins. Scale bars = 25 μm.

To confirm the biologic relevance of WISP-1 in PSC biology, we first returned to the *in vivo* residence of PSC, in the perivascular niche of human adipose tissue. By immunohistochemical detection in human adipose tissue, WISP-1 showed a strong perivascular localization in both the perivascular cells and associated vascular wall connective tissue (Fig. 1). WISP-1 immunoreactivity of intermediate to strong intensity was present in vessels of all calibers within adipose tissue, including arterioles, capillaries, venules and larger veins. In contrast, inconspicuous staining of adipocytes or other tissue elements was seen. These findings were supported by the Human Protein Atlas (proteinatlas.org), which confirmed predominant WISP-1 immunoreactivity in the vascular wall in sections of adipose tissue. Also published within the Human Protein Atlas was high WISP-1 immunoreactivity in the tunica media and adventitia of thick walled arteries (CAB012205, Soft tissue 2, proteinatlas.org), which not present for analysis on our study material. Thus, WISP-1 protein is highly expressed across vessel types in human adipose tissue, in a distribution that includes both presumptive microvascular pericytes and adventitial progenitor cells (APCs).

**Figure 2:**
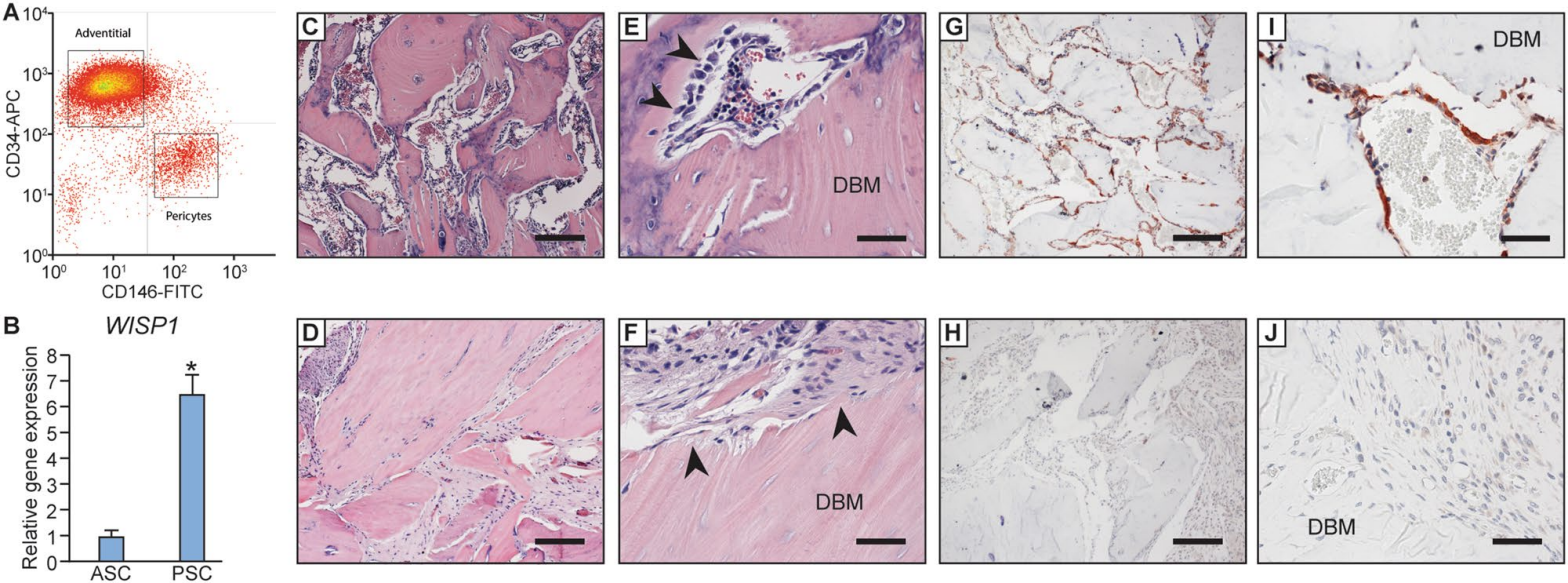
WISP-1 expression in human PSC after FACS isolation and upon orthopaedic transplantation. (**A**) Fluorescence activated cell sorting (FACS) of human lipoaspirate was performed per established protocols to isolate perivascular stem/stromal cells (PSC). After isolation of CD31−CD45− non-endothelial/non-hematopoietic cells (*not shown*), PSC were further defined as adventitial progenitor cells (APC, CD34+CD146−) or microvascular pericytes (CD146+CD34−). (**B**) Quantitative RT-PCR for *WISP1* transcripts, examined in passage one PSC or unpurified ASC (adipose-derived stromal cells) from the same patient sample. Performed in technical triplicate. (**C**–**F**) H&E appearance of rat spine fusion segments. (**C**,**E**) H&E appearance of PSC-treated spinal fusion segments with new bone formation, incorporation of demineralized bone matrix (DBM), and conspicuous bone-lining osteoblasts (arrowheads). (**D**,**F**) H&E appearance of control-treated spinal fusion segments, with little new bone formation, DBM particles without connections to each other and embedded in fibrous stroma, and inactive bone lining cells (arrowheads). (**G**–**J**) WISP-1 indirect immunohistochemical staining of PSC treated spine fusion segments. WISP-1 immunoreactivity appears brown, while nuclear Hematoxylin counterstain appears blue. (**G**,**I**) Strong WISP-1 immunoreactivity in bone lining cells among PSC treated samples. (**H**,**J**) Weak to intermediate WISP-1 immunoreactivity in inflammatory cells and stromal fibroblasts among control treated samples. *P < 0.05. Scale bars (parts **C**,**D**,**G**,**H**) 25 µm; Scale bar (parts **E**,**F**,**I**,**J**) 100 µm.

Next, perivascular stem/stromal cells (PSC) were FACS purified from human lipoaspirate using previously validated methods to isolate microvascular pericytes (CD146+CD34−CD31−CD45) and APCs (CD34+CD146−CD31−CD45−)(Fig. 2A). After removal of CD31+ endothelial and CD45+ inflammatory cells (representing 38.15% of total mononuclear cells), adventitial progenitor cells (54.82%) and microvascular pericytes (4.36%) were collected and combined to constitute PSC. Next, qRT-PCR for *WISP1* transcripts was assayed between PSC and unpurified, culture defined ASC (adipose-derived stromal cells) from the same patient sample (Fig. 2B). In agreement with prior findings, *WISP-1* gene expression was elevated among purified PSC (>6 fold enrichment of *WISP1* transcripts among PSC).

Finally, FACS purified, uncultured PSC were applied to a previously validated lumbar spinal fusion model in athymic rats, and WISP-1 expression was interrogated during the process of *in vivo* bone formation^20^(Fig. 2C–J). In this xenotransplant model, and as we have previously reported, defined human PSC cell numbers (1.5 × 10^6^ human cells) when applied in a demineralized bone matrix scaffold to the lateral aspects of the rat lumbar spine induces a 100% incidence of spinal fusion after a 4 week period^20^. Spinal fusion segments were treated with or without human PSC (see Table 1 for treatment group allocation). The H&E appearance of PSC treated spinal fusion segments demonstrates interconnected bone trabeculae which incorporate the devitalized bone graft scaffold (DBM, demineralized bone matrix) (Fig. 2C,E). Higher magnification demonstrates easily discernable bone-lining osteoblasts within PSC treated samples (arrowheads, Fig. 2E), as well as vascular and bone marrow elements between bony trabeculae. In contrast, spinal fusion segments treated without PSC showed DBM particles predominantly embedded in fibrous tissue and with only sporadic new bone formation (Fig. 2D). At higher magnification, control treated sections showed inconspicuous bone lining cells were present on the edges of DBM particles (arrowheads), which lacked the cuboidal morphology of synthetically active osteoblasts. WISP-1 immunolocalization was next performed on representative PSC- or control-treated spinal fusion segments (Fig. 2G–J). In PSC-treated specimens, strong and diffuse WISP-1 immunoreactivity was seen in bone lining osteoblasts (Fig. 2G,I), as well as foci of endochondral ossification (*not shown*). In contrast, weak to intermediate and more focal immunostaining for WISP-1 was identified in control sections, predominantly in inflammatory cells and stromal fibroblasts (Fig. 2H,J). In aggregate, high WISP-1 expression was observed among cells of the perivascular niche of adipose tissue, both *in situ*, after FACS purification, and upon *in vivo* orthopaedic application.

**Table 1:** Animal allocation.

Treatment group	Scaffold	Cell #	Animal #
Scaffold alone	Demineralized bone matrix putty	—	5
FACS-derived PSC		1.5 × 10^6^ PSC	6

### WISP-1 knockdown and PSC differentiation

**Figure 3:**
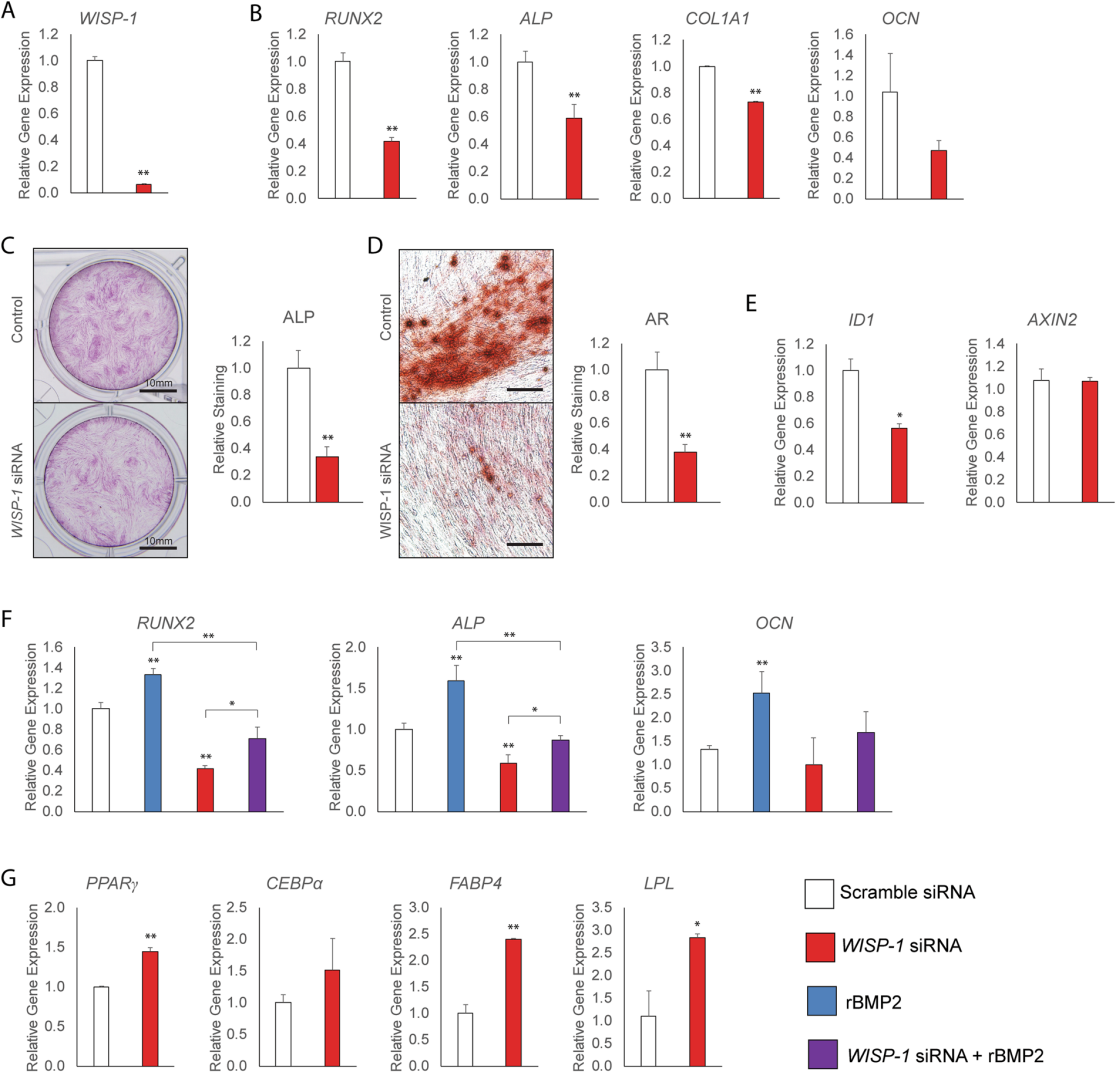
Osteogenic and adipogenic differentiation of human PSC with *WISP-1* knockdown. *WISP-1* siRNA or scramble siRNA treated PSC were evaluated for osteogenic and adipogenic differentiation. (**A**) Knockdown efficiency of *WISP-1* in human PSC, assessed by qRT-PCR. (**B**–**D**) Osteogenic differentiation of human PSC with or without *WISP-1* knockdown. (**B**) Expression levels of osteogenic gene markers by qRT-PCR at 3 days of osteogenic differentiation, including *RUNX2 (Runt-related transcription factor 2), ALP (Alkaline Phosphatase), COL1A1 (Type I Collagen)*, and *OCN* (*Osteocalcin*). (**C**) Alkaline phosphatase (ALP) staining and photometric quantification after 12 days of osteogenic differentiation with or without *WISP-1* knockdown. (**D**) Bone nodule formation examined by Alizarin red (AR) staining after 12 days of osteogenic differentiation with or without *WISP-1* knockdown. (**E**) Expression levels of markers of BMP and Wnt signaling activity by qRT-PCR at 3 days of osteogenic differentiation with or without *WISP-1* knockdown, including *ID1 (Inhibitor of DNA binding 1)* and *AXIN2 (Axis Inhibition Protein 2)*. (**F**) PSC were treated with rBMP2 (50 ng/mL), with or without *WISP-1* knockdown. Expression levels of osteogenic gene markers by qRT-PCR at 3 days of osteogenic differentiation, including *RUNX2*, *ALP*, and *OCN*. (**G**) Adipogenic differentiation of human PSC with or without *WISP-1* knockdown. Adipocytic gene markers assessed by quantitative RT-PCR at 3 days of differentiation, including *PPAR*γ (*Peroxisome proliferator-activated receptor gamma*), *CEBP*α (*CCAAT/enhancer-binding protein alpha*), *FABP4 (Fatty acid binding protein 4)*, and *LPL* (*Lipoprotein lipase*). *P < 0.05; **P < 0.01. Scale bars (part **C**) 10 mm; scale bars (part **D**) 200 µm.

We turned to the *in vitro* consequences of *WISP-1* knockdown in human PSC differentiation using siRNA. Knockdown efficiency using *WISP-1* siRNA was 93.4% in comparison to scramble siRNA, as assessed by qRT-PCR (Fig. 3A). The consequences of *WISP-1* knockdown on human PSC osteogenic differentiation were next assessed (Fig. 3B–D). Consistent with prior reports in other cell types, gene expression for markers of osteogenic differentiation were significantly reduced among PSC with *WISP-1* knockdown (Fig. 3B). This included a reduction in the transcription factor *runt-related transcription factor 2 (RUNX2*, 58.4% reduction), the enzyme *alkaline phosphatase* (*ALP*, 41.3% reduction), the principal matrix protein of bone *collagen type I alpha I chain (COL1A1*, 27.0% reduction), as well as a non-significant reduction in the terminal differentiation marker *osteocalcin* (*OCN*, 57.2% reduction) (Fig. 3B). In agreement with gene expression studies, staining and photometric quantification of ALP enzymatic activity was significantly reduced with *WISP-1* knockdown (66.5% reduction, d 12 of differentiation) (Fig. 3C). Bone nodule deposition was assessed by Alizarin Red (AR) staining and photometric quantification, and likewise showed a robust inhibition of bone nodules with *WISP-1* knockdown (62.1% reduction, d 12 of differentiation) (Fig. 3D).

As mentioned, WISP-1 is known to positively regulate both canonical BMP and canonical Wnt signaling in the process of osteogenic differentiation. In the context of *WISP-1* knockdown in human PSC, gene markers of BMP and Wnt signaling activity were next assessed (Fig. 3E). Three days post-transfection, the BMP2 responsive element, *inhibitor of DNA binding 1 (ID1*) was significantly reduced in expression under *WISP-1* siRNA treatment conditions (44.1% reduction). In contrast, transcript abundance for the canonical Wnt signaling marker *AXIN2 (Axis Inhibition Protein 2)* was not significantly affected by *WISP-1* knockdown. These results suggested that at least in the context of *WISP-1* knockdown in human PSC osteogenic differentiation, differential BMP signaling may represent the dominant effect. To further pursue this, recombinant BMP2 (rBMP2) was added to osteogenic differentiation medium (ODM), with or without *WISP-1* knockdown (Fig. 3F). As expected, rBMP2 treatment under control conditions increased transcripts for osteogenic markers, including a 33.2–119.2% increase in *RUNX2, ALP* and *OCN* at 3 days differentiation (blue bars, Fig. 3F). *WISP1* knockdown again significantly reduced all osteogenic gene markers (red bars, Fig. 3F). Moreover, *WISP-1* knockdown significantly reversed rBMP2 induction of osteogenic marker expression (purple bars, Fig. 3F).

Next, the consequences of *WISP-1* knockdown on adipogenic differentiation of human PSC was assessed (Fig. 3G). Three days after induction of adipogenic differentiation, adipocytic gene expression was assayed by qRT-PCR. All markers assayed were significantly increased by *WISP-1* knockdown, including *PPARy (peroxisome proliferator-activated receptor gamma*, 44.0% increase*), CEBPa (CCAAT/enhancer-binding protein alpha*, 51.2% increase*), FABP4 (fatty acid binding protein 4*, 139.8% increase*)*, and *LPL* (*lipoprotein lipase*, 172.8% increase*)* (Fig. 3G). In summary, knockdown of *WISP-1* by siRNA diminished PSC osteogenic differentiation, but also led to an enhancement of adipocytic gene expression in PSC.

### WISP-1 gain of function and PSC differentiation

**Figure 4:**
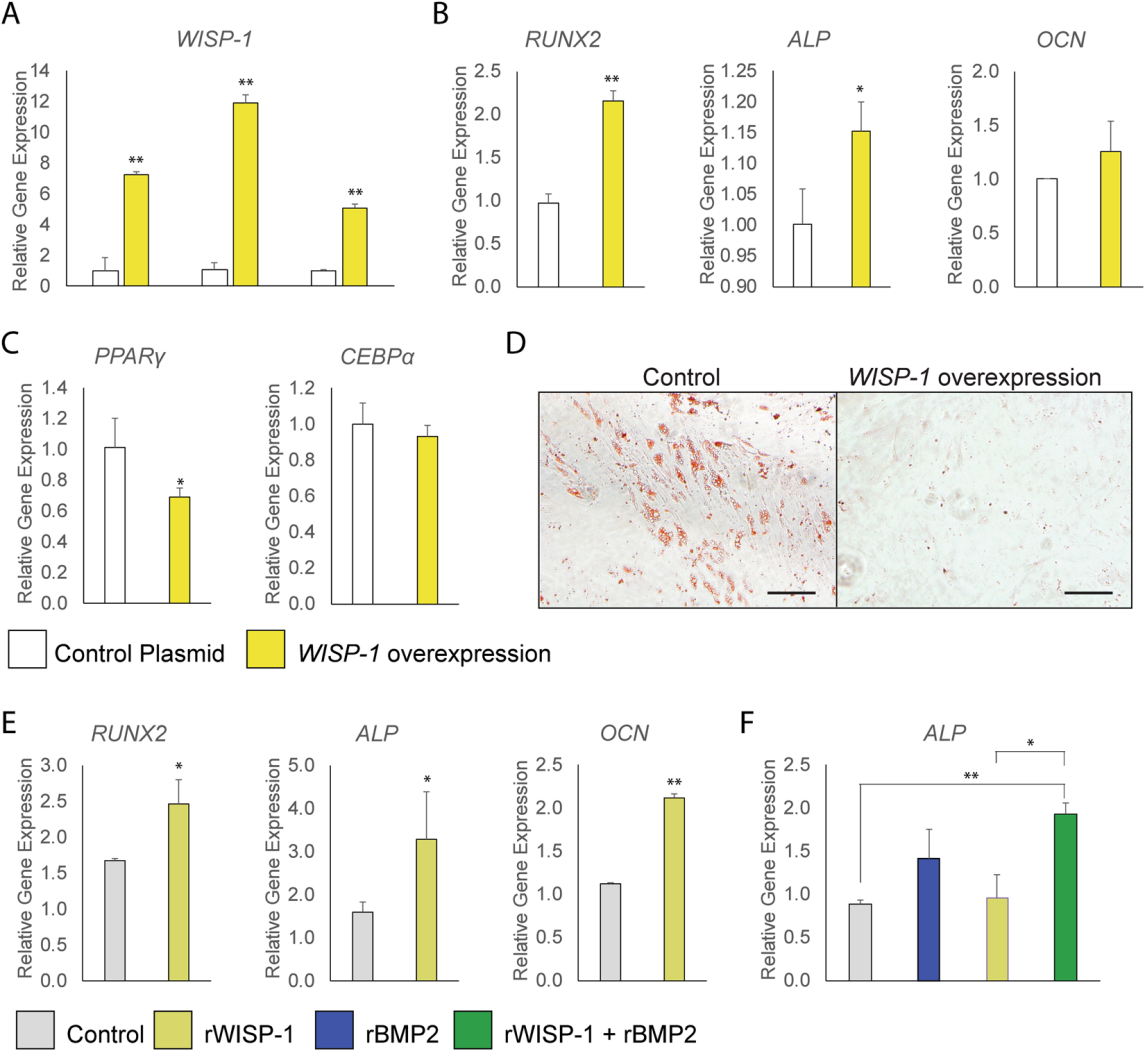
Osteogenic and adipogenic differentiation of human PSC with *WISP-1* overexpression or rWISP-1 protein. (**A**–**D**) *WISP-1* overexpression or control plasmid treated PSC were evaluated for osteogenic and adipogenic differentiation. (**A**) Efficacy of *WISP-1* plasmid in human PSC, assessed by qRT-PCR at 6, 9, and 15 days. (**B**) Expression levels of osteogenic gene markers by qRT-PCR at 3 days of osteogenic differentiation, including *RUNX2 (Runt-related transcription factor 2), ALP (Alkaline Phosphatase), COL1A1 (Type I Collagen)*, and *OCN* (*Osteocalcin*). (**C**) Adipocytic gene markers assessed by quantitative RT-PCR at 3 days of differentiation, including *PPAR*γ (*Peroxisome proliferator-activated receptor gamma*), *CEBP*α (*CCAAT/enhancer-binding protein alpha*). (**D**) Oil red O staining of human PSC with or without *WISP-1* overexpression, 15 days of differentiation. (**E**,**F**) Osteogenic differentiation of human PSC with or without recombinant (r)WISP-1 protein (200 ng/mL). (**E**) Expression levels of osteogenic gene markers by qRT-PCR at 3 days of osteogenic differentiation, including *RUNX2*, *ALP*, and *OCN*. (**F**) Effects of rWISP-1 (200 ng/mL) and rBMP2 (50 ng/mL) combined application in human PSC. Expression levels of *ALP* assessed by qRT-PCR at 3 days of osteogenic differentiation. *P < 0.05; **P < 0.01. Scale bars: 100 µm.

The effects of gain-of-function in WISP-1 signaling were next assessed, using either *WISP-1* overexpression or recombinant WISP-1 protein (Fig. 4). First, PSC were transfected using a *WISP-1* expression plasmid and the consequences on osteogenesis and adipogenesis were examined (Fig. 4A–D). As expected, PSC transfection led to a significant upregulation in the production of *WISP-1* transcripts which was maintained across three timepoints of osteogenic differentiation (days 6, 9, and 15) (Fig. 4A). *WISP-1* overexpression in PSC under osteogenic differentiation conditions led to an increase in osteogenic gene markers, including *RUNX2* (119.1% increase), *ALP* (15.1% increase), and a non-significant trend toward increased *OCN* expression (Fig. 4B). *WISP-1* overexpression was next examined in PSC under adipogenic differentiation conditions (Fig. 4C,D). *WISP-1* overexpression led to a significant reduction in adipogenic gene markers, including *PPARγ* (42.0% reduction) and a trend towards reduced *CEBPα* expression (Fig. 4C). Oil Red O staining likewise showed a significant reduction in the intracellular accumulation of lipid with *WISP-1* overexpression after 15 days differentiation (Fig. 4D).

PSC osteogenic differentiation was next assessed using recombinant (r)WISP-1 protein (200 ng/mL). In agreement with *WISP-1* overexpression studies, rWISP-1 induced a significant increase in osteogenic gene marker expression, including *RUNX2* (77.9% increase), *ALP* (183.4% increase), and *OCN* (89.8% increase) (Fig. 4E). The potential combinatorial effect of rBMP2 and rWISP-1 on osteogenic differentiation was next examined (Fig. 4F). A significant increase in *ALP* expression was observed with rBMP2 alone (blue bar, Fig. 4F). rWISP-1 co-application with rBMP2 led to an increase in *ALP e*xpression over rBMP2 (green bar, Fig. 4F). In summary, WISP-1 signaling was observed to exert pro-osteogenic/anti-adipocytic effects in human PSC.

## Discussion

Here, we have elucidated a new role of WISP-1 signaling within stromal progenitor cells of the perivascular niche of human adipose tissue. WISP-1 has been previously defined by its role as a bone matricellular protein and in its positive regulation of osteoblastogenic differentiation in other osteoblastic cell types^13,19^. However, prior studies have described a role for WISP-1 in the vascular wall, specifically in vascular smooth muscle cell proliferation and migration^21^. Importantly, vascular smooth muscle cells represent a distinct and terminally differentiated cell, and lack the multipotentiality of PSC. WISP-1 has also been implicated in the promotion of angiogenesis, especially in tumor associated angiogenesis^22–24^. In the current study, we found enrichment of WISP-1 within PSC both *in situ*, after purification, and after *in vivo* application in a bone defect microenvironment. This led us to examine the modulation of PSC differentiation by WISP-1 signaling.

WISP-1 has previously been shown to induce osteogenic differentiation by multiple mechanisms, including association with biglycan (BGN) in the matrix of mineralizing tissues^25^, activation of BMP signaling^18^, and potentiation of canonical Wnt signaling^13^. Our examination of PSC *in vitro* suggested a role for WISP-1 in the osteogenic differentiation of PSC by regulation of BMP activity. Previous examination of the effects of WISP-1 on BMP2 activity by Ono *et al*. showed that a direct interaction between BMP2 and WISP-1 is essential for the enhancement of canonical BMP signaling, and that neutralizing antibodies that block integrin α_5_β_1_ mitigated this interaction^18^. An independent study by Kohaha *et al*. found that BMP2 and WISP-1 had synergistic effects in an ectopic bone formation model in mice^17^. In the current study, we observed that the knockdown of *WISP-1* led to a marked decrease in transcripts of the BMP responsive element *ID1*. Moreover *WISP-1* knockdown antagonized the pro-osteogenic effects of BMP2, and recombinant WISP-1 synergized with rBMP2 with the promotion of PSC osteogenesis.

The effects of WISP-1 on adipogenic differentiation are less well understood. Recent studies have found that human ASC express and secrete WISP-1 during *in vitro* adipogenesis, and that changes in adiposity alter WISP-1 expression^26^. As a regulator of both Wnt and BMP signaling, it would be anticipated that the role of WISP-1 in adipogenic differentiation is complex. Our results of the anti-adipocytic effects of WISP-1 are an independent confirmation of a recent report by Ferrand *et al*. who observed that WISP-1 is a negative regulator of adipogenesis^27^. Here, they observed that WISP-1 expression is reduced during adipogenesis, that adipocytic cell lines demonstrate a significant reduction in adipogenesis with *WISP-1* overexpression associated with decreased PPARγ transcriptional activity, and that *WISP-1* knockdown induces preadipocyte differentiation^27^. The current study confirms these same anti-adipocytic effects of WISP-1 in human multipotent mesenchymal cells.

In summary, our experiments showed that the matricellular protein WISP-1 is highly expressed in PSC in the vascular wall of human adipose tissue. Our results demonstrate the importance of WISP-1 in the positive regulation of osteogenesis and negative regulation of adipogenesis in human stem cell types within the perivascular niche. Thus, manipulation of WISP-1 signaling may have broad and important therapeutic implications for either fields of bone or adipose tissue engineering. In the context of bone tissue engineering, a cellular therapy augmented with WISP-1 protein may be used to ossify bones with more success and rapidity than an unstimulated cell type. Conversely, antagonism of WISP-1 or downstream signaling may be of future benefit when an adipocytic cell fate is most desirable in soft tissue augmentation or reconstruction.

## Materials and Methods

### Perivascular stem/stromal (PSC) cell isolation

PSC were isolated from human subcutaneous adipose tissue via fluorescence activated cell sorting (FACS). Human lipoaspirate was obtained from healthy adult donors under IRB approval at UCLA and JHU with a waiver of informed consent, and was stored for less than 48 h at 4 °C before processing. The SVF of human lipoaspirate was obtained by collagenase digestion. Briefly, lipoaspirate was diluted with an equal volume of phosphate-buffered saline (PBS) and digested with Dulbecco’s modified Eagle’s medium (DMEM) containing 3.5% bovine serum albumin (Sigma-Aldrich, St. Louis) and 1 mg/ml type II collagenase for 70 min under agitation at 37 °C. Adipocytes were separated and removed by centrifugation. The cell pellet was resuspended in red blood cell lysis buffer (155 mM NH_4_Cl, 10 mM KHCO3, and 0.1 mM EDTA) and incubated at room temperature. After centrifugation, cells were resuspended in PBS and filtered at 70 μm. The resulting SVF was further processed for cell sorting, using a mixture of the following directly conjugated antibodies: anti-CD34-allophycocyanin (1:100; BD Pharmingen, San Diego, CA), anti-CD45-allophycocyanin-cyanin 7 (1:100; BD Pharmingen), anti-CD146-fluorescein isothiocyanate (1:100; Bio-Rad, Hercules, CA), and anti-CD31-allophycocyanin-cyanin 7 (1:100, Bio Legend, San Diego, CA (summary of antibodies presented in Table 2). All incubations were performed at 4 °C for 15 min. The solution was then passed through a 70-μm cell filter and then run on a FACS Diva 8.0.1 cell sorter (BD Biosciences). In this manner, microvessel pericytes (CD146+CD34−CD45−CD31−) and adventitial cells (CD34+CD146−CD45−CD31−) were isolated and combined to constitute the PSC population. Sorted PSC were either snap frozen for RNA isolation, applied in a rat spinal fusion model, or culture expanded for *in vitro* studies. For *in vitro* expansion, cells were cultured at 37 °C in a humidified atmosphere containing 95% air and 5% CO_2_. PSC were cultured in DMEM, 20% fetal bovine serum (FBS), 1% penicillin/streptomycin. Medium was changed every three d unless otherwise noted.

**Table 2:** Antibodies used in fluorescence activated cell sorting.

Antibody	Fluorochrome	Company-Catalog #
Mouse Anti-Human CD34	APC	BD Pharmingen-555824
Mouse Anti-Human CD146	FITC	Bio Rad-MCA2141F
Mouse Anti-Human CD45	APC-cy7	BD Pharmingen-557833
Mouse Anti-Human CD31	APC-cy7	Bio Legend-303119

### Osteogenic differentiation assays

Assays for PSC differentiation are adapted from our prior publications^28,29^. Osteogenic differentiation medium (ODM) was constituted with 10 mM β-glycerophosphate and 50 μM ascorbic acid in DMEM + 20% FBS. In select experiments, ODM was supplemented with recombinant WISP-1 (200 ng/mL) (ab50041, Abcam, Cambridge MA) or BMP2 (50 ng/mL, Medtronic), and protein supplementation was changed every three d of differentiation. In select experiments, *WISP-1* gene expression was manipulated by siRNA or overexpression by plasmid using appropriate controls (see below).

Alkaline phosphatase staining was performed using the Leukocyte Alkaline Phosphatase Kit (Sigma-Aldrich). Briefly, cells were seeded in 24 well plates at a density of 1 × 10^4^ cells/well. Cells were cultured under osteogenic differentiation conditions for 12 d prior to staining. Cells were then washed with PBS and fixed with formalin for 10 min at room temperature. Following fixation, cells were stained using Leukocyte Alkaline Phosphatase Kit (Sigma-Aldrich) according to the manufacturer’s protocol. Cells were incubated in alkaline phosphatase stain for 15 min at 37 °C, then washed with PBS. Cells were allowed to dry and pictures were taken at 100x magnification using Olympus IX71 inverted system microscope (Olympus, Cypress, CA). Relative staining was quantified using Adobe Photoshop CC2015.

For the detection of mineralization, cells were seeded in growth medium in 24 well plates at a density of 1 × 10^4^ cells/well. 24 h after seeding cells, basal medium was replaced with ODM in triplicates per each treatment for 12 d. Cells were washed with PBS and fixed with 4% paraformaldehyde. Following fixation, cells were stained with 2% alizarin red (Sigma-Aldrich, Saint Louis, Missouri) at room temperature for 15 min then washed with deionized water and allowed to dry. Pictures were taken at 100x magnification using Olympus IX71 inverted system microscope (Olympus, Cypress, CA). In order to quantify bone nodule deposition, 10% v/v acetic acid was added and cells were incubated at room temperature for 30 min with shaking. Cells were then scraped from the wells and vortexed for 30 s. Next, cells were overlaid with mineral oil and heated to 85 °C for 10 min. Briefly, cells were cooled on ice for 5 min then centrifuged at 20,000 × g for 15 min. 10% ammonium hydroxide was added to adjust the pH to between 4.1 and 4.15. Absorbance was measured in triplicates at 405 nm in 96 well plates using Epoch microspectrophotometer (Bio-Tek, Winooski, VT).

### Adipogenic differentiation assays

PSC were seeded in six well plates at a density of 1 × 10^5^ cells per well and allowed to adhere overnight. Medium was then replaced with Mesencult Adipogenic Differentiation medium (StemCell technologies Inc., Vancouver, BC). Cells were cultured under adipogenic differentiation conditions for fifteen d for gene expression analysis. Differentiation medium was changed every three d. For visualization by Oil Red O staining: After 15 d of adipogenic differentiation, cells were washed with PBS, and fixed with 10% formalin for 30 min. Oil Red O stock solution was prepared from powder (Sigma, St. Louis, MO) by mixing 300 mg Oil Red O powder with 100 ml of 99% isopropanol. Stock solution was diluted 3:2 stock solution: deionized water and allowed to sit at room temperature for 10 min. The working solution was then filtered by gravity filtration. After fixation by formalin, cells were washed with water and 60% isopropanol was added to wells for 5 min before staining. Oil Red O working solution was then added to each well and incubated at 37 °C for 30 min. Following incubation, the cells were washed with tap water images were taken using Q capture software.

### Ribonucleic acid (RNA) isolation and quantitative real-time polymerase chain reaction (qRT-PCR)

Gene expression was assayed by quantitative RT-PCR, based on our previous methods^29,30^. Primers sequences are shown in Table 3. Timepoints for specific gene expression include 3–15 d of osteogenic or adipogenic differentiation, with additional details provided in the accompanying figure legends. Briefly, total RNA was extracted using RNEasy Kit (Qiagen, Santa Clarita, CA). 1 μg of total RNA from each sample was subjected to first-strand complementary deoxyribonucleic acid (cDNA) synthesis using the SuperScript III Reverse-Transcriptase Kit (Life Technologies) to a final volume of 20 μL. The reverse transcription reaction was performed at 65 °C for 5 min, followed by 50 °C for 50 min and 85 °C for 5 min. For qRT-PCR, the reaction was performed using 2 × SYBR green RT-PCR master mix and an ABI PRISM 7300 qRT-PCR system instrument (Applied Biosystems, Foster City, CA). qRT-PCR was performed using 96 well optical plates at 95 °C for 10 min, followed by 40 cycles at 95 °C for 15 s, and at 60 °C for 60 s. The relative quantification of gene expression was performed using a Comparative CT method according to the manufacturer’s protocol and was normalized to the expression levels of *ACTB* in each sample.

**Table 3:** Quantitative RT-PCR primers.

Gene	Forward	Reverse
*ACTB*	5′-CTGGAACGGTGAAGGTGACA-3′	5′-AAGGGACTTCCTGTAACAATGCA-3′
*ALP*	5′-GACCCTTGACCCCCACAAT-3′	5′-GCTCGTACTGCATGTCCCCT-5′
*AXIN2*	5′-CAAGGGCCAGGTCACCAA-3′	5′-CCCCCAACCCATCTTCGT-3′
*CEBPα*	5′-TGGACAAGAACAGCAACGAGTA-3′	5′-ATTGTCACTGGTCAGCTCCAG-3′
*COL1A1*	5′-TACCCCACTCAGCCCAGTGT-3′	5′-ACCAGACATGCCTCTTGTCCTT-3′
*FABP4*	5′-ACGAGAGGATGATAAACTGGTGG-3′	5′-GCGAACTTCAGTCCAGGTCAAC-3′
*ID1*	5′-ACGACATGAACGGCTGTTACTCAC-3′	5′-CTCCAACTGAAGGTCCCTGATGTAG-3′
*LPL*	5′-TTGCAGAGAGAGGACTCGGA-3′	5′-GGAGTTGCACCTGTATGCCT-3′
*OCN*	5′-AGCAAAGGTGCAGCCTTTGT-3′	5′-GCGCCTGGGTCTCTTCACT-3′
*PPARγ*	5′-GGGGTGATGTGTTTGAACTTG-3′	5′-GACAGGAAAGACAACAGACAAATC-3′
*RUNX2*	5′-ATGGCGGGTAACGATGAAAAT-3′	5′-ACGGCGGGGAAGACTGTGC-3′
*WISP-1*	5′-GTATGTGAGGACGACGCCAAG-3′	5′-GGCTATGCAGTTCCTGTGCC-3′

### Small interfering RNA (siRNA) and transfection

Knockdown of *WISP-1* was performed using Silencer Select chemically synthesized siRNA (Thermo Fisher Scientific, catalog number: 4392420; S16873). Cells were seeded in 6 well plates at a density of 4 × 10^4^/well. At 50% confluence, basal medium was replaced with antibiotic-free basal medium. Transfection was performed using X-tremeGENE siRNA Transfection Reagent (Sigma-Aldrich) and 150 pM *WISP-1* siRNA or scramble siRNA diluted in minimal essential medium (Opti-MEM). For the confirmation of siRNA efficiency: six h post-transfection, medium was replaced with basal medium and the efficiency of the knockdown was validated using qRT-PCR. For alizarin red (AR) and alkaline phosphatase (ALP) staining: Six h post-transfection, medium was replaced with ODM and cells were cultured under osteogenic differentiation conditions for up to 12 d.

### WISP-1 overexpression

*WISP-1* overexpression was assayed using human *WISP-1* ORF mammalian expression plasmid (HG10442, Sino Biological, North Wales, PA). 24 h prior to transfection, cells were seeded in 6 well plates at 60% confluence. For transfection, 1 μg of DNA was mixed with 3 μl of Roche Xtreme gene HP transfection reagent in 100 μL of Opti-MEM and incubated at room temperature for 30 min. The DNA/Transfection reagent mixture was then added dropwise to wells. Cell lysate was measured for *WISP-1* gene expression to confirm efficacy of the plasmid.

### *In vivo* spinal fusion assay

A rat posterolateral lumbar spinal fusion assay with human PSC treatment was performed with adaptations from our previous methods under University of California, Los Angeles IACUC approval and with methods carried out in accordance with institutional guidelines and regulations^20^. Implantation of human PSC utilized a demineralized bone matrix (DBX) carrier (300 μl per side of spine; Musculoskeletal Transplant Foundation, Edison, NJ). DBX is a combination of morselized cortical and cancellous bone chips mixed with sodium hyaluronate. 1.5 × 10^6^ human PSC in 50 μl PBS were mixed mechanically with each 300 μl implant, and kept on ice prior to implantation. Animal allocation is shown in Table 1.

Posterolateral lumbar spinal fusion was performed on 8-week-old athymic rats as previously described^20^. Animals were housed and experiments were performed in accordance with guidelines of the Chancellor’s Animal Research Committee of the Office for Protection of Research Subjects at the University of California, Los Angeles. All animals were treated with postoperative medications of buprenorphine for 48 h and trimethoprim/sulfamethoxazole for 10 d, for pain management and prevention of infection, respectively. Rats were anesthetized using isoflurane (5% induction, 2–3% maintenance). Posterior midline incisions were made over the caudal portion of the lumbar spine, and two separate fascial incisions were made 4 mm bilaterally from the midline. Blunt muscle splitting technique was used lateral to the facet joints to expose the transverse processes of L4 and L5 lumbar spines. The processes were then decorticated using a low speed burr under regular irrigation with sterile saline solution to cool the decortication site and maintain a clean surface for implantation. Next, the treatment material was delivered via a scaffold, implanted between the transverse processes bilaterally into the paraspinal muscle bed. Finally, the fasciae and skin were each closed using a simple continuous technique with 4-0 Vicryl sutures (Ethicon Endo-Surgery, Blue Ash, OH).

Rats were sacrificed 4 weeks postsurgery via CO_2_ overdose, and the spines were harvested for analysis. Spinal fusion with PSC treatment was confirmed by biomechanical testing and radiographic analysis as previously reported^20^. Samples were next decalcified using 19% EDTA and embedded in paraffin. Immunohistochemical staining was performed with primary antibodies against WISP-1 (1:100, ab155654, Abcam, Cambridge, MA) using the ABC (Vector Laboratories, Burlingame, CA) method. Immunohistochemistry was performed after paraffin sections were deparaffinized, rehydrated, rinsed, and incubated with 3% H_2_O_2_ for 20 min. All sections were then blocked with 0.1% bovine serum albumin in PBS for 1 h. At a dilution of 1:100, primary antibodies were added to each section and incubated at 37 °C for 1 h at 4 °C. Images were obtained on an Olympus (Center Valley, PA) BX51 microscope.

### Statistical analysis

All results were expressed as mean ± standard deviation (SD). Statistical analyses were performed using the SPSS16.0 software. All data were normally distributed. Student’s t test was used for two-group comparisons, and one-way ANOVA test was used for comparisons of 3 or more groups, followed by Tukey’s *post hoc* test. Differences were considered significant when *P < 0.05 and *P < 0.01.

## Data Availability

The datasets generated or analyzed during the current study are available from the corresponding author on reasonable request.
